# Fault Detection and Classification in MMC-HVDC Systems Using Learning Methods

**DOI:** 10.3390/s20164438

**Published:** 2020-08-08

**Authors:** Qinghua Wang, Yuexiao Yu, Hosameldin O. A. Ahmed, Mohamed Darwish, Asoke K. Nandi

**Affiliations:** 1 School of Mechatronic Engineering, Xi’an Technological University, Xi’an 710021, China; wqhhuazi@163.com; 2 College of Engineering, Design and Physical Sciences, Brunel University London, Uxbridge UB8 3PH, UK; yuyuexiao333@hotmail.com (Y.Y.); hosameldin.ahmed2@brunel.ac.uk (H.O.A.A.); mohamed.darwish@brunel.ac.uk (M.D.); 3 State Grid Sichuan Electric Power Research Institute of China, Chengdu 610094, China

**Keywords:** MMC-HVDC, fault detection, fault classification, CNN, AE-based DNN, SoftMax classifier, classification accuracy, speed

## Abstract

In this paper, we explore learning methods to improve the performance of the open-circuit fault diagnosis of modular multilevel converters (MMCs). Two deep learning methods, namely, convolutional neural networks (CNN) and auto encoder based deep neural networks (AE-based DNN), as well as stand-alone SoftMax classifier are explored for the detection and classification of faults of MMC-based high voltage direct current converter (MMC-HVDC). Only AC-side three-phase current and the upper and lower bridges’ currents of the MMCs are used directly in our proposed approaches without any explicit feature extraction or feature subset selection. The two-terminal MMC-HVDC system is implemented in Power Systems Computer-Aided Design/Electromagnetic Transients including DC (PSCAD/EMTDC) to verify and compare our methods. The simulation results indicate CNN, AE-based DNN, and SoftMax classifier can detect and classify faults with high detection accuracy and classification accuracy. Compared with CNN and AE-based DNN, the SoftMax classifier performed better in detection and classification accuracy as well as testing speed. The detection accuracy of AE-based DNN is a little better than CNN, while CNN needs less training time than the AE-based DNN and SoftMax classifier.

## 1. Introduction

With the increasing application of modular multilevel converter-based high-voltage direct current (MMC-HVDC) systems, the reliability of MMC is of major importance in ensuring power systems are safe and reliable. Topology configuration redundant strategies of fault-tolerant systems are useful methods to improve reliability, which can be achieved by using more semiconductor devices as switches in an SM [1] or integrating redundant SMs into the arm submodule [2]. However, it is crucial that fault detection is a precondition for fault-tolerant operation, which is required to be as fast and accurate as possible, to ensure converter continuous service. Therefore, fault detection and classification are among the challenging tasks in MMC-HVDC systems in improving its reliability and, thus, reducing potential dangers in the power systems, because there are a large number of power electronic sub-modules (SMs) in the MMC circuit, and each SM is a potential failure point [3,4].

The research of fault detection and classification in MMC-HVDC systems applications can be broadly categorized into three basic approaches that are mechanism-based, signal processing-based, and artificial intelligence-based [5]. All the mechanism-based methods need many sensors monitoring the inner characteristics (circulating current, arm currents, capacitor voltages, etc.). Signal processing-based methods employ output characteristics rather than inner characteristics to detect a fault. Signal processing-based methods have been deemed reliable and fast by researchers [6,7,8,9], with the advancement of signal processing methods in recent years. However, both of them need suitable methods to obtain expected inner characteristics or threshold of certain derived features, such as zero-crossing current slope or harmonic content, which degrades the robustness of fault detection and classification. The learning methods do not need any input of mathematical models of MMC functionality and any threshold setting; yet, they can improve the accuracy of fault diagnosis due to their advantage of nonlinear representations.

Neural networks have been used by many researchers. Khomfoi and Tolbert [10] propose a fault diagnosis and reconfiguration technique for a cascaded H-bridge multilevel inverter drive using principal component analysis (PCA) and neural network (NN). In this method, the genetic algorithm is used to select valuable principal components. Simulation and experimental results showed that the proposed method is satisfactory to detect fault type, fault location, and reconfiguration. Wang et al. [11] propose an artificial NN-based robust DC fault protection algorithm for MMC high voltage direct current grid. In this work, the discrete wavelet transform has been used as an extractor of distinctive features at the input of the ANN. Furqan et al. [12] present NN-based fault detection and diagnosis system for three-phase inverter using several features extracted from the Clarke transformed output as an input of NNs. Merlin et al. [13] design thirteen artificial NNs for the voltage-source converter-HVDC systems to detect a fault condition in the whole HVDC system, based only on voltage waveforms measured at the rectifier substation.

Although the NN based methods have achieved some improvements in the diagnosis of failed converters and identification of defective switches [14,15], the prerequisite for the successful application of NNs is to have enough training data and long training time. Multi-class relevance vector machines (RVM) and support vector machine (SVM) replace a neural network to classify and locate the faults, because of their rapid training speed and strongly regularized characteristic [5]. Wang et al. [16] use a PCA and multiclass RVM approach for fault diagnosis of cascaded H-bridge multilevel inverter system. Wang et al. [17] propose and analyze a fault-diagnosis technique to identify shorted switches based on features generated through the wavelet transform of the converter output and subsequent classification in SVMs. The multi-class SVM is trained with multiple recordings of the output of each fault condition, as well as the converter under normal operation. Jiao et al. [18] used the three-phase AC output side voltage of MMC as the fault characteristic signal, combined with PCA data preprocessing and firefly algorithm optimized SVM (FA-SVM) for MMC fault diagnosis. Zhang and Wang [19] propose a least-squares-based ɛ-support vector regression scheme, which captures fault features via the Hilbert–Huang transform. Fault features are used as the inputs of ɛ-support vector regression to obtain fault distance. Then, the least-squares method is utilized to optimize the parameters of the model, so that it can meet the demand on fault location for MMC–MTDC transmission lines.

To build the aforementioned artificial intelligence machine, feature extraction techniques such as Fourier analysis [20,21], wavelet transform [14,15], Clarke transform [12] or feature subset selection techniques, such as principal component analysis (PCA) [10,22] and multidimensional scaling (MDS), plays an important role. Sometimes to select suitable sub-features, the genetic algorithm (GA) [10,22,23] or particle swarm optimization (PSO) [24] are employed. It is well known that feature extraction has always been a bottleneck in the field of fault diagnosis. Moreover, the feature extraction and all the following post-operations increase the computation burden.

Deep learning methods have been explored to learn the features from the data, which can be generalized to different cases. Zhu et al. [25] proposed convolutional neural networks (CNN) for fault classification and fault location in AC transmission lines with back-to-back MMC-HVDC, in which two convolutional layers were used to extract the complex features of the voltage and the current signals of only one terminal of transmission lines. Kiranyaz et al. [26] use 1-D CNN to detect and localize the switch open-circuit fault using four cell capacitor voltage, circulating current and load current signals. This method can achieve a detection probability of 0.989 and an average identification probability of 0.997 in less than 100 ms. Qu et al. [27] propose CNN for MMC fault detection using each capacitor’s voltage signal. Wang et al. [28] propose CNN for DC fault detection and classification using wavelet logarithmic energy entropy of transient current signal. In the past our research group proposed some related methods of NNs [29,30,31], AE-based DNN [32], and SoftMax classifier [33] for bearing fault detection and classification, but not for MMC-HVDC. Moreover, to the best of our knowledge, the use of deep learning methods for MMC fault detection and classification have been very limited, and there is no comparison of two deep learning methods. Furthermore, afore-mentioned CNNs have achieved success, but their advantages have not been explored completely, e.g., the ability of feature extraction, the speed of processing, and its stability. In summary, up to now, there is still much room for further improvement of performance of the open-circuit fault diagnosis of MMCs.

To address this and achieve high fault classification accuracy with fewer sensors and reduced computational time for fault diagnosis of MMCs, we propose two deep learning methods and one stand-alone SoftMax classifier for MMCs faults detection and classification using raw data collected from current sensors to recognize automatically the open-circuit failures of IGBT in MMCs. The contributions of this paper are as follows:

- Only current sensors data are used for fault diagnosis and achieved high accuracy of fault detection and classification.
- Multichannel current signals are used instead of a single channel to improve reliability, because the sensors may have some faults.
- Excellent accuracies of fault detection and identification without data preprocessing or post-operations are achieved;
- Two deep learning methods and a stand-alone SoftMax classifier are used with raw data collected by current sensors, to achieve improved classification accuracy and reduced computation time.
- Performance comparison of CNN, AE-based DNN, and SoftMax Classifier in terms of fault diagnosis accuracy, stability, and speed for MMC-HVDC fault diagnosis are provided. This paper is organized as follows. Section 2 introduces the topology and data acquisition from MMC. Section 3 proposes the framework of this paper and the design of CNN, AE-based DNN, and SoftMax classifier. The feasibility and performance of the proposed approaches are evaluated in Section 4. Section 5 compares the three deep learning methods. Conclusions are drawn in Section 6.

## 2. MMC Topology and Data Acquisition

**Figure 1:**
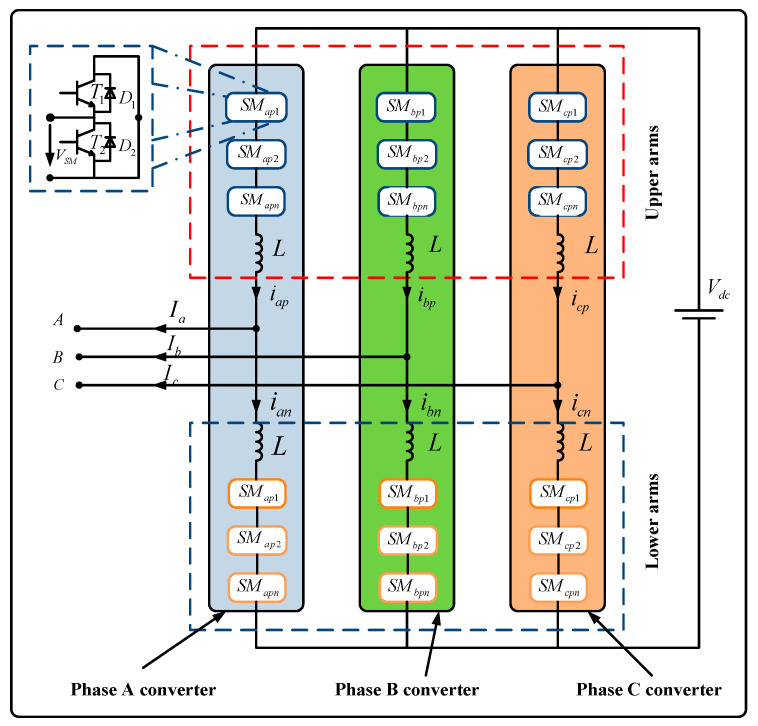
Structure of a three-phase modular multilevel converters (MMC) with half-bridge submodules.

The data for this study were simulated from a two-terminal model of the MMC-HVDC transmission power system using PSCAD/EMTDC [34]. It solves the differential equations of the entire power system and its controls. Figure 1 shows that each phase of the three-phase MMC consists of two arms (upper and lower) that are connected to two inductors L. Each arm contains a series of SMs, and each SM involves two IGBTs (i.e., T_1_ and T_2_), two diodes D, and a DC storage capacitor.

**Figure 2:**
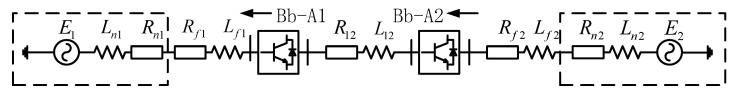
Structure of the high voltage direct current converter (HVDC).

In our simulation (Table 1), we recorded 9 channels of data for normal and 6 different locations of IGBT break-circuit fault manually for each bridge (namely A-phase lower SMs, A-phase upper SMs, B-phase lower SMs, B-phase upper SMs, C-phase lower SMs, and C-phase upper SMs). There are seven MMC health conditions (Table 2) and 100 cases of IGBT break-circuit faults occurring at different IGBTs of the six bridges at different times. The power system is depicted in Figure 2. The type of SMs is half-bridge and the direction of the flow is shown as the arrow above. Ba-A1 and Ba-A2 are two AC bus bars. Bb-A1 and Bb-A2 are two DC bus bars. E1 is an equivalent voltage source for an AC network. E2 is a wind farm.

The total time period used is 0.1 s, while the time for the IGBT open circuit fault duration is varied from 0.03 to 0.07 s. The simulation time step is 2 μs and the sampling frequency is 20 μs. The acquired data channels for fault diagnosis are AC-side three-phase current (*I*_a_, *I*_b_, *I*_c_) and three-phase circulation current (I_diffa_, I_diffb_, I_diffc_).

## 3. The Framework of Fault Classification and Design of Deep Learning Methods

### 3.1. The Framework for Fault Detection and Classification

**Figure 3:**
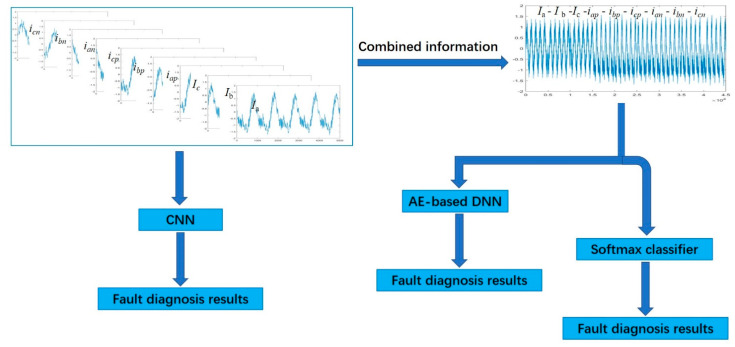
Framework for fault detection and classification for MMC.

This paper proposes three methods to complete both the fault detection and classification task for MMC, as shown in Figure 3, which are CNN, AE-based DNN, and a stand-alone SoftMax classifier. CNN processes the raw sensors data, which are nine current signals (*I*_a_, *I*_b_, *I*_c_, *i_ap_*, *i_bp_*, *i_cp_*, *i_an_*, *i_bn_*, and *i_cn_*) and obtains the fault diagnosis results. AE-based DNN and SoftMax process the combined information that is concatenated the measurements of these nine parameters, to form a vector of samples that represent the current health condition of the MMCs, and then obtain the fault diagnosis results.

### 3.2. Design of CNN

Convolutional neural networks (CNNs) are widely used tools for deep learning which is different from the traditional feed-forward ANN, because of its three architectural properties of the visual cortex cell: local receptive regions, shared weights, and subsampling. The crucial advantage of CNNs is that both feature extraction and classification operations are fused into a single machine learning body to be jointly optimized to maximize the classification performances [26].

**Figure 4:**
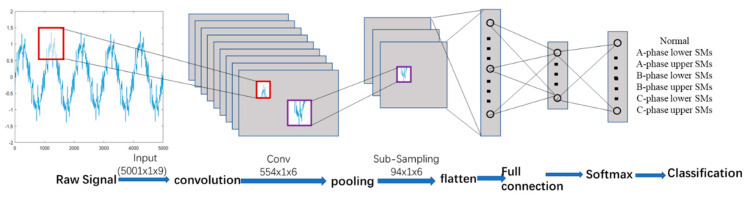
Architecture of the signal-level CNN classifier.

CNN consists of multiple layers, such as Figure 4, which are the input layer, convolutional layer, activation layer, pooling layer, full connect layer, SoftMax layer, and a classification layer. Among these layers, there are two basic layers in CNN, which are the convolutional layer and the pooling layer. Convolution operation implements the first two properties that are local receptive regions and shared weights. The pooling operation implements the subsampling property [35].

A convolutional layer consists of neurons that connect to small regions of the input and operate the convolution computation. The output feature map of the convolutional layer can be written as:

(1) $$F_{j}=\varphi \left(\sum_{i=1}^{N}W_{i,j}⊗I_{i}+b_{j}\right),$$

For the *j*th filter, the output is a new feature map $F_{j}$, Where $W_{i,j}$ and $b_{j}$ denote the **j**th filter kernel and bias, respectively, $I_{i}$ is the input matrix of the *i*th channel, ⊗ represents the convolutional operation, and $I_{i}$ is convoluted with a corresponding filter kernel $W_{i,j}$. The sum of all convolved matrices is then obtained and a bias term $b_{j}$ is added to each element of the resulting matrix. There are several choices we could make activation function φ be a non-linear. However, in this paper, we simply use a named leaky rectified linear unit (leaky ReLU). The function of leaky ReLU is given by:

(2) $$\varphi \left(x\right)=\{\begin{array}{ll} x, & \;x\geq 0\\ scale*x, & \;x<0 \end{array}$$

It is a simple threshold that makes the negative value be zero. Then, we can obtain the output feature map, $F_{j}$.

Pooling layers perform down-sampling operations. Pooling functions usually include max-pooling and average-pooling. In this paper, the average-pooling function is applied which outputs the average values of rectangular regions of its input. In a fully connected layer, neurons between two adjacent layers are fully pairwise connected but neurons within the same layer share no connections. Then, the SoftMax function is commonly adopted for classification tasks. The introduction of SoftMax will be presented in the following Section 3.4.

### 3.3. Design of AE-Based DNN

**Figure 5:**
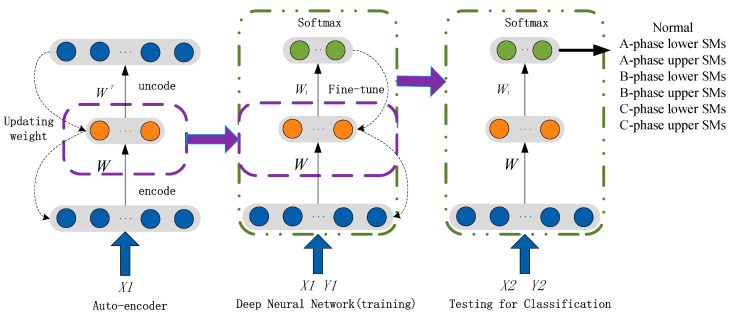
Architecture of the autoencoder (AE)-based deep neural network (DNN).

An AE-based deep neural network (DNN) is constructed by several autoencoders (AEs), stacked with each other and a SoftMax classifier on the output layer. In this paper, we stacked one AE with a SoftMax classifier, as can be seen in Figure 5. The AE needs to be pretrained by Greedy layer-wise training algorithm. The simplest form of an AE includes three layers: the input layer, hidden layer, and output layer. An AE network consists of an encoder and a decoder. The encoder maps the input to a hidden representation and the decoder attempts to map this representation back to the original input. Given an unlabeled vector sample x, the encoder network can be explicitly defined as:

(3) $$h=f\left(w_{1}x+b_{1}\right),$$

Similarly, the decoder network can be defined as:

(4) $$\hat{x}=g\left(w_{2}x+b_{2}\right),$$

where $\hat{x}$ is the approximate reconstruction of the inputs, and θ={w,b} is the reconstructing parameters, and *f* and g are the activation function of the encoder and decoder, respectively. The reconstruction error E between the inputs *x* and output $\hat{x}$ is defined as:

(5) $$E=\underset{mean\;squared\;error}{\underbrace{\frac{1}{N}\;\overset{N}{\underset{i=1}{\sum }}{\left(x_{i}-{\hat{x}}_{i}\right)}^{2}}}\;+\lambda *\underset{regularization}{\underset{L_{2}}{\underbrace{\Omega_{weights}}}}$$

where the first part is the mean square variance used to measure the average discrepancy and *N* is the number of neurons in the output layer, and the second part is the regularization term used to prevent overfitting, and *λ* is the coefficient for the *L*_2_ regularization term.

(6) $$\Omega_{weights}=\frac{1}{2}\overset{L}{\underset{l}{\sum }}\overset{N}{\underset{j}{\sum }}{\left(w_{j}^{\left(l\right)}\right)}^{2}$$

where L is the number of hidden layers. The following subsection introduces the SoftMax classifier.

### 3.4. Introduction of SoftMax Classifier

The SoftMax function, also known as softargmax or normalized exponential function, is a function that takes as input a vector of K real numbers and normalizes it into a probability distribution consisting of K probabilities proportional to the exponentials of the input numbers. It is calculated as:

(7) $$y_{r}\left(x\right)=P\left(c_{r}|x,\theta \right)=\frac{exp\left(a_{r}\left(x\right)\right)}{\sum_{j=1}^{k}exp\left(a_{j}\left(x\right)\right)},$$

The loss function can use mean squared error function and the cross-entropy function. In this paper, we used the cross-entropy function, which is given by:

(8) $$E=-\sum_{i=1}^{N}\sum_{j=1}^{k}t_{ij}lny_{ij},$$

where $t_{ij}$ is the indicator that the *i*th example belongs to the *j*th class, $y_{ij}$ is the output for example *i*, which here is the value from the SoftMax function.

## 4. Experimental Study

Seven conditions of MMCs status have been recorded which include normal, A-phase lower SMs, A-phase upper SMs, B-phase lower SMs, B-phase upper SMs, C-phase lower SMs, and C-phase upper SMs faults. A total of 100 examples were collected from each condition. Thus, there are a total of 700 (100 × 7) raw data files to process with. All the nine parameters, i.e., *I*_a_, *I*_b_, *I*_c_, *i_ap_*, *i_bp_*, *i_cp_*, *i_an_*, *i_bn_*, and *i_cn_*, were recorded to obtain 5001-time samples.

Experiments were conducted for testing data proportions from 0.1 to 0.9 and 20 run times for each testing data proportion. Testing data proportion is the ratio of the number of test samples to the total number of samples. We need to point out that the detection and classification results in the following paper are the average of 20 run results. In order not to be influenced by the differences in data used, it is important to ensure that these methods work with the same data at each run. The following code is pseudo-code, which can explain this scenario.

*For* TestingDataProportion = 0.1:0.1:0.9

  *For* i = 1:20

   [trainData testData] = split(RawData, TestingDataProportion);

   CNN = trainCNN(trainData);

   ResultsCNN = CNN(testData);

   [trainDataCI testDataCI] = combined Information(trainData, testData);

   AE-basedDNN = trainAE-basedDNN(trainDataCI);

   ResultsAE = AE-basedDNN(testDataCI);

   SoftMax = trainSoftMax(trainDataCI);

   ResultsSoftMax = SoftMax(testDataCI)

  *End*

*End*

### 4.1. Implementation Details and Results of CNN

#### 4.1.1. Implementation Details of CNN

Figure 4 illustrates the architecture of CNN for fault detection and classification. The input data is the raw sensor signals. Each channel denotes one sensor, which records 5001-time samples. Therefore, the size of input current signals is [5001 × 1 × 9], where the length is 5001 and the height is 1, as the signals are one dimensional, and the depth is 9, as the signals come from 9 channels. The input is convolved with 6 filters of size 1 x 30 with stride 9 and padding 3, then applied a leaky ReLU function, in which the scalar multiplier for negative inputs is set as 0.01, resulting in a new feature map of size 554 × 1 and 6 channels. The sequence is pooling operation, which is applied to each feature map separately. Our pooling size is set 6 × 1 and stride is 6. Therefore, a convolution feature map is divided into several disjoint patches and then the average value in each patch is selected to represent the patch and transmit to the pooling layer, then the feature map is reduced to 94 × 1 by the pooling operation.

As stochastic gradient descent with momentum (SGDM) algorithm may reduce the oscillations along the path of the steepest descent towards the optimum that is sometimes caused by stochastic gradient descent algorithm [36], we use the SGDM algorithm to update the parameters of the deep NN. The stochastic gradient descent with momentum update is

$$\theta_{l+1}=\theta_{l}-\alpha \nabla E\left(\theta_{l}\right)+\gamma \left(\theta_{l}-\theta_{l-1}\right)$$

where l stands for the iteration number, θ is the parameter vector, α is the learning rate, ∇E(θ) is the gradient of the loss function, and γ determines the contribution of the previous gradient step to the current iteration. Here, we set the momentum γ at 0.95, the learning rate α at 0.01, and the maximum number of epochs to use for training at 30.

#### 4.1.2. Results of CNN

The accuracy of the CNN fault detection is shown in Table 3. For fault detection, the output network is divided into two types: fault and normal. We can see from Table 3, when the testing proportion is 0.1~0.5 and 0.7, the detection accuracy is 100%. The minimum of the detection accuracy is 99.7% at the testing proportion of 0.9. There are 0.3% of fault cases misclassified as normal cases.

**Figure 6:**
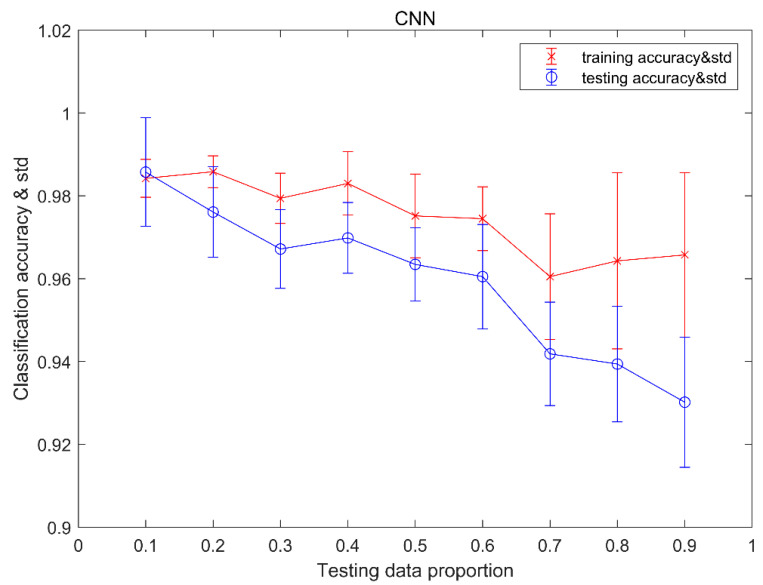
The classification accuracy and the standard deviation of CNN.

The classification results of training and testing data using convolutional NNs are shown in Figure 6. From the viewpoint of trending, we can see that, with the testing data proportion increases, both classification accuracy for training data and testing data decline. For the training dataset, the standard deviation of classification accuracy increases with the increase of the testing data proportion. For testing data set, the maximum of mean accuracy is 98.6% with testing data proportion of 0.1 and the minimum of the average accuracy is 93.0% with testing data proportion of 0.9. The standard deviation of classification accuracy in the middle of the testing data proportion is smaller than both ends of the testing data proportion. Moreover, for each testing data proportion, the standard deviation of classification accuracy for the training data set is less than the standard deviation of classification accuracy for the testing data set.

Table 4 provides a confusion matrix of the classification results for each condition with testing data proportions of 0.2, 0.5, and 0.8. As can be seen from Table 4, the recognition of the normal condition of the MMCs is 100%, with 0.2, 0.5, and 0.8 testing data proportions. With a 0.2 testing data proportion, our method misclassified 3.2% of testing examples of condition 4 as condition 2, and 2% of testing examples of condition 4 as condition 6. With a 0.5 testing data proportion, our method misclassified 1.6% of testing examples of condition 4 as condition 2, and 3.4% of testing examples of condition 4 as condition 6. Furthermore, with 0.8 testing data proportion, our method misclassified 0.8% of testing examples of condition 4 as condition 2, and 6.4% of testing examples of condition 4 as condition 6.

### 4.2. Implementation Details and Results of AE-Based DNN

#### 4.2.1. Implementation Details of AE-Based DNN

First, the measurements of nine current signals were concatenated to form a vector of samples that represent the current health condition of the MMCs. This gave a total of 45,009 (5001 × 9) samples dimension for each vector of health condition. Second, we used the AE with three layers: the input layer, hidden layer, and output layer, in which, the number of neurons in the hidden layer is set as 250, which means the sample dimension will be reduced from 45,009 to 250. An AE network consists of an encoder and a decoder. The transfer function for the encoder and the decoder is the Satlin function and the logistic sigmoid function, respectively. Satlin function is a positive saturating linear transfer function given as:

(9) $$f\left(z\right)=\{\begin{array}{cc} 0, & if\;z\leq 0\\ z, & if\;0<z<1\\ 1, & if\;z\geq 1 \end{array},$$

**Figure 7:**
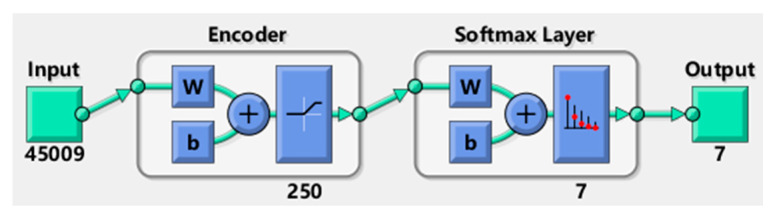
The structure of AE-based DNN.

The algorithm used for training the autoencoder applied scaled conjugate gradient descent (SCGD). The maximum number of training epochs for this autoencoder is set as 10. Third, the 250 features achieved by trained AE are used as the input of the SoftMax classifier. The maximum number of training epochs for the SoftMax classifier is set as 20. Next, we stacked the trained AE and SoftMax classifier into a deep NN. Finally, we trained this deep NN using the training data. The structure of deep net is shown in the Figure 7. The maximum number of epochs for training this deep net is set to 1000.

#### 4.2.2. Results of AE-Based DNN

The fault detection results of the AE -based DNN are shown in Table 5. When the testing proportion varies from 0.1 to 0.7, the detection accuracy is 100%. The lowest detection accuracy is 99.7% at the testing proportion of 0.9. There are 0.3% fault cases misclassified as normal cases. Compared with Table 3 of CNN, AE-based DNN has better detection accuracy.

**Figure 8:**
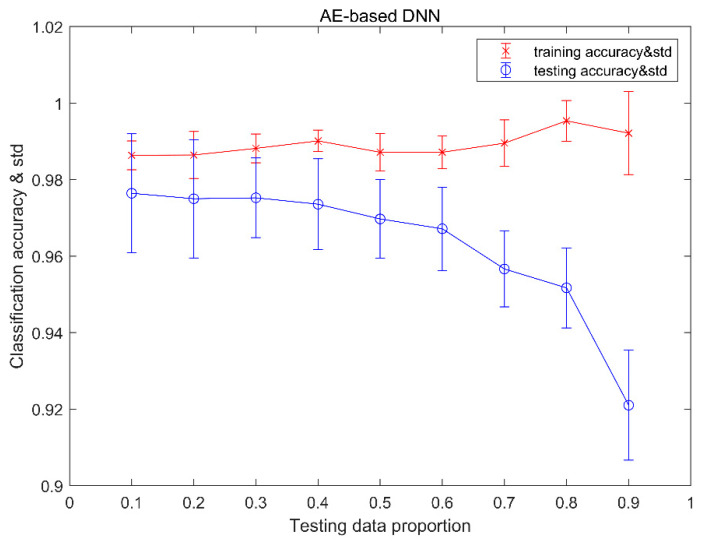
The classification accuracy and the standard deviation of AE-based DNN.

Figure 8 shows the classification results of training and testing data using AE-based DNN. From the viewpoint of trending analysis, we can see that with the testing data proportion increase, the classification mean accuracy for testing data declines, but the classification accuracy for training data increases. For the training data set, the highest average accuracy is 99.5%, with a testing data proportion of 0.8, and the lowest is 98.6%, with a testing data proportion of 0.1. The standard deviation of classification accuracy increases with the increase of the testing data proportion. For the testing data set, the max of mean accuracy is 97.6%, with the testing data proportion of 0.1, and the minimum of mean accuracy is 92.1%, with a testing data proportion of 0.9. The standard deviation of classification accuracy in the middle of the testing data proportion is smaller than both ends of the testing data proportion. We can also see that, for each testing data proportion, the standard deviation of classification accuracy for the training data set is less than the standard deviation of classification accuracy for the testing data set.

Table 6 provides a confusion matrix of the classification results for each condition with testing data proportions of 0.2, 0.5, and 0.8. As can be seen from Table 6, the recognition of the normal condition of the MMCs is 100%, with 0.2, 0.5, and 0.8 testing data proportions. With a 0.2 testing data proportion, our method misclassified 1.5% of testing examples of condition 3 as condition 5. With a 0.5 testing data proportion, our method misclassified 1.8% of testing examples of condition 3 as condition 5, and 0.2% of testing examples of condition 3 as condition 7. With a 0.8 testing data proportion, our method misclassified 0.7% of testing examples of condition 3 as condition 4, 1.6% of testing examples of condition 3 as condition 5, 1% of testing examples of condition 3 as condition 6, and 1.9% of testing examples of condition 3 as condition 7.

### 4.3. Results of SoftMax Classifier

The accuracy of SoftMax classifier fault detection is shown in Table 7. The detection accuracy is 100% at all testing proportions.

**Figure 9:**
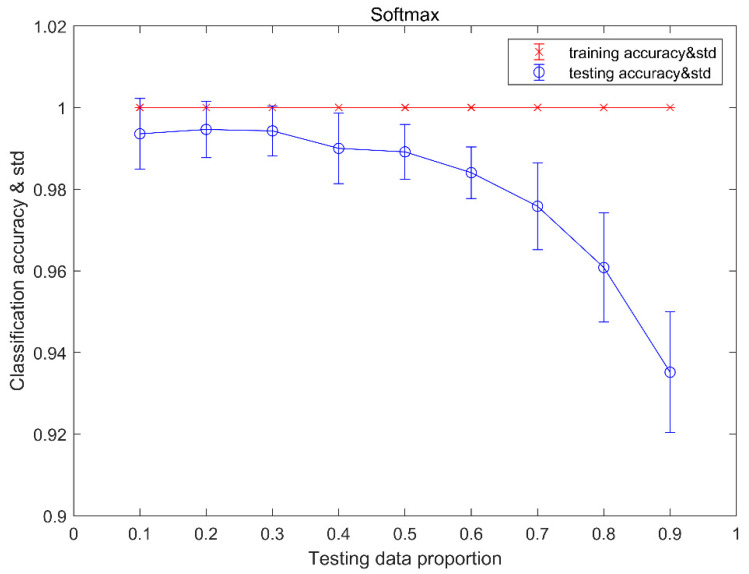
The classification accuracy and the standard deviation of the stand-alone SoftMax classifier.

Figure 9 shows the classification results of training and testing data using the SoftMax classifier. From the trending view, we can see that, with the testing data proportion increases, the classification average accuracy for testing data declines, but the classification average accuracy for training data keeps steady at 100%. The standard deviation of classification accuracy in the middle of the testing data proportion is smaller than both the end of testing data proportion for testing data set, but the standard deviation of classification accuracy is 0. For testing data set, the highest average accuracy is 99.5%, with a testing data proportion of 0.2, and the lowest average accuracy is 93.5%, with a testing data proportion of 0.9. It is obvious to see that for each testing data proportion the standard deviation of classification accuracy for training data set is less than the standard deviation of classification accuracy for testing data set.

Table 8 provides a confusion matrix of the classification results for each condition with testing data proportions of 0.2, 0.5, and 0.8. As can be seen from Table 8, the recognition of the normal condition of the MMCs is 100% with 0.2, 0.5, and 0.8 testing data proportions. With a 0.2 testing data proportion, our method misclassified none of the testing examples of condition 4. With a 0.5 testing data proportion, our method misclassified 0.4% of testing examples of condition 4 as condition 2. With a 0.8 testing data proportion, our method misclassified 1.5% of testing examples of condition 4 as condition 2, and 1.9% of testing examples of condition 4 as condition 6.

Above all, for the training data set, with the increase of testing data proportion, the average accuracy of SoftMax keeps steady which is 100% and the average accuracy of CNN decreases, but the average accuracy of AE-based increases. The standard deviation of accuracy for SoftMax keeps steady at 0, and the standard deviation of accuracy for other methods increases with the increase of the testing data proportion. For the testing data set, the average accuracy of all methods decreases, with the increase of the testing data proportion and the standard deviation of accuracy in the middle being less than both ends of the testing data proportion for all methods.

## 5. Comparisons

**Figure 10:**
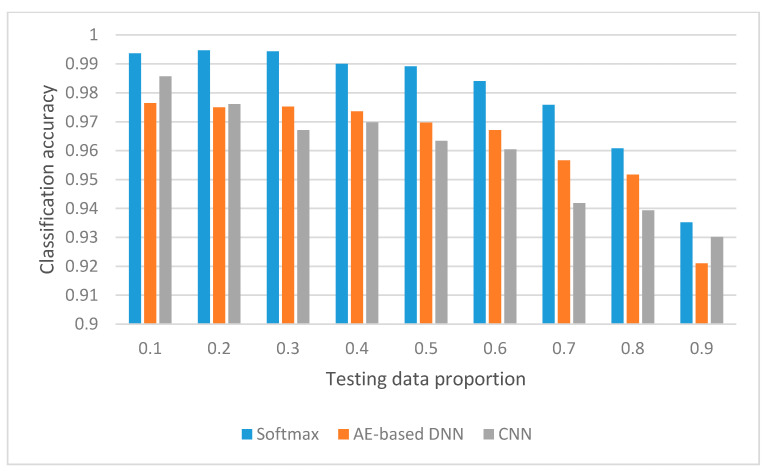
Comparison of classification accuracy for the three methods.

**Figure 11:**
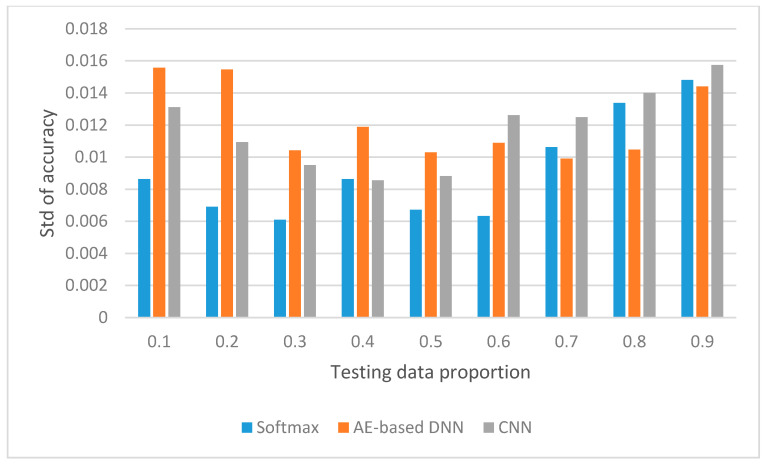
Comparison of the standard deviation of classification accuracy for the three methods.

**Figure 12:**
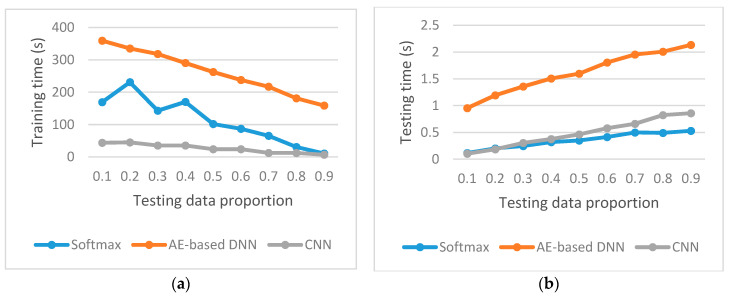
Comparison of speed for the three methods. (**a**) Comparison of training time spent by the three methods, and (**b**) comparison of testing time spent by the three methods.

We have compared the three methods on the classification accuracy and the standard deviation of classification accuracy for the testing data with the testing data proportion from 0.1 to 0.9, and compared the three methods from the viewpoint of training time spent and testing time spent, which are presented in Figure 10, Figure 11 and Figure 12, respectively.

### 5.1. Comparison of Average Accuracy

From Figure 10, we can see that the SoftMax classifier behaves outstandingly on the testing data proportion from 0.1 to 0.9 compared to CNN and AE-based DNN. When the testing data proportion is 0.1, 0.2, and 0.9, which locates both ends, the classification accuracy of CNN is better than the AE-based DNN.

### 5.2. Comparison of Standard Deviation

We know that, in statistics, the standard deviation is a measure that is used to quantify the amount of variation or dispersion of a set of data values. A low standard deviation indicates that the values tend to be close to the expected value of set, while a high standard deviation indicates that the values are spread out over a wider range. From Figure 11, it is clear that the standard deviation of accuracy of SoftMax is lower than other methods when the testing data proportions are in the range of 0.1 to 0.6. This implies that, for every run for different training data set and testing data set, the classification accuracy of SoftMax is more stable and other methods are more spread out. When the testing data proportion varies from 0.7 to 0.9, the AE-based DNN has the lowest standard deviation. AE-based DNN is the most spread out when the testing data proportion is from 0.1 to 0.5, and CNN is the most spread out when the testing data proportion is from 0.6 to 0.9.

### 5.3. Speed Comparison

Figure 12 describes the training time and testing time spent by three methods. It shows that, for each testing data proportion, the AE-based DNN spends more training time than other methods, and the CNN spends the least training time, and the SoftMax takes the least testing time when the testing data proportion varies from 0.3 to 0.9, and the AE-based DNN spends the most testing time.

For CNN, the testing time is 0.48 s for 70 examples (testing data proportion of 0.1) and 4.08 s for 630 examples (testing data proportion of 0.9), i.e., the average testing time per example is 0.007 s. For AE-based DNN, the testing time is 0.95 s for 70 examples (testing data proportion of 0.1) and 2.1 s for 630 examples (testing data proportion of 0.9); so, the average testing time per example is 0.004 s. For the SoftMax Classifier, the testing time is 0.11 s for 70 examples (testing data proportion of 0.1) and 0.53 s for 630 examples (testing data proportion of 0.9), i.e., the average testing time per example is 0.001 s. Please note that the time spent is not only to detect fault but also to classify the kind of faults.

In these experiments, the stand-alone SoftMax classifier provides better functionality, including fault detection accuracy, classification accuracy, least standard deviation, speed, as well as its strong ability in dealing with high dimensional data. The AE-based DNN has the second best classification ability, but it needs more training time and testing time. CNN has enough classification accuracy, and it needs the least training time.

## 6. Conclusions

Fault detection and classification are two of the challenging tasks in MMC-HVDC systems. This paper presented two deep learning methods (CNN and AE-based DNN) and a stand-alone SoftMax classifier for fault detection and classification. CNN and AE-based DNN can fuse both feature extraction and classification operations into a single machine learning scheme for joint optimization, to maximize the classification performance, which avoided the design of handcrafted features. In this paper, we only use raw current sensor data as input to our proposed approaches to detect and classify faults of MMC-HVDC. The simulation results in PSCAD/EMTDC show that three methods all have a high detection accuracy of more than 99.7%. The stand-alone SoftMax classifier has the best detection accuracy (100%), while AE-based DNN performs a little better than of CNN. Three methods also have high classification accuracy, small standard deviation, and high speed. SoftMax classifier is better than others in classification accuracy and testing speed, while CNN needs the least training time.

## Figures and Tables

**Table 1:** Parameters of MMC.

Parameters	Value
number of SMs per arm	9
SM capacitor	1000 uF
arm inductance	50 mH
AC frequency	50 Hz

**Table 2:** MMC health conditions.

Faulty Bridge	Label Value
Normal	1
A-phase lower SMs	2
A-phase upper SMs	3
B-phase lower SMs	4
B-phase upper SMs	5
C-phase lower SMs	6
C-phase upper SMs	7

**Table 3:** Fault detection accuracy of convolutional neural networks (CNN).

Testing Data Proportion	0.1	0.2	0.3	0.4	0.5	0.6	0.7	0.8	0.9
Detection accuracy (%)	100	100	100	100	100	99.9	100	99.8	99.7

**Table 4:** Sample confusion matrix of the classification results of CNN.

**(a) Testing Data Proportion = 0.2.**							
	**Normal**	**A-phase lower SMs**	**A-phase upper SMs**	**B-phase lower SMs**	**B-phase upper SMs**	**C-phase lower SMs**	**C-phase upper SMs**
Normal	100	0	0	0	0	0	0
A-phase lower SMs	0	97.8	0	3.2	0	0	0
A-phase upper SMs	0	0	97.3	0	0	0	0.8
B-phase lower SMs	0	0.7	0	94.8	0	2.2	0
B-phase upper SMs	0	0	2.2	0	99.8	0	3.2
C-phase lower SMs	0	0.7	0	2	0	97.8	0
C-phase upper SMs	0	0.2	0.5	0	0.2	0	96
**(b) Testing data proportion = 0.5.**							
	**Normal**	**A-phase lower SMs**	**A-phase upper SMs**	**B-phase lower SMs**	**B-phase upper SMs**	**C-phase lower SMs**	**C-phase upper SMs**
Normal	100	0	0	0	0	0	0
A-phase lower SMs	0	95	0	1.6	0.2	0.7	1.3
A-phase upper SMs	0	0	97.2	0	0.9	0	1.1
B-phase lower SMs	0	1	0	95	0	1.9	0
B-phase upper SMs	0	0	1.9	0	96.1	0	3
C-phase lower SMs	0	3.6	0.2	3.4	0.3	96.9	0.4
C-phase upper SMs	0	0.4	0.7	0	2.5	0.5	94.2
**(c) Testing data proportion = 0.8**							
	**Normal**	**A-phase lower SMs**	**A-phase upper SMs**	**B-phase lower SMs**	**B-phase upper SMs**	**C-phase lower SMs**	**C-phase upper SMs**
Normal	100	0.2	0	0	0	0.5	1
A-phase lower SMs	0	91.6	0	0.8	0	0.9	2.3
A-phase upper SMs	0	0	94.4	0	2.5	0	2.2
B-phase lower SMs	0	3.8	0.4	92.8	0	0.6	0.2
B-phase upper SMs	0	0	3.2	0	90.8	0	2.5
C-phase lower SMs	0	4	0.3	6.4	0.6	97.1	0.9
C-phase upper SMs	0	0.4	1.7	0	6.1	0.9	90.9

**Table 5:** Detection accuracy of AE-based DNN.

Testing Data Proportion	0.1	0.2	0.3	0.4	0.5	0.6	0.7	0.8	0.9
Detection accuracy	100	100	100	100	100	100	100	99.9	99.7

**Table 6:** Sample confusion matrix of the classification results of AE-based DNN.

**(a) Testing data proportion = 0.2**							
	**Normal**	**A-phase lower SMs**	**A-phase upper SMs**	**B-phase lower SMs**	**B-phase upper SMs**	**C-phase lower SMs**	**C-phase upper SMs**
Normal	100	0	0	0	0	0	0
A-phase lower SMs	0	97	0	3.2	0	1.5	0.3
A-phase upper SMs	0	0	98.5	0	0	0	0
B-phase lower SMs	0	0.7	0	95.5	0	1	0.2
B-phase upper SMs	0	0	1.5	0	97	0	2.5
C-phase lower SMs	0	2.3	0	1.3	0.5	97.5	0
C-phase upper SMs	0	0	0	0	2.5	0	97
**(b) Testing data proportion = 0.5**							
	**Normal**	**A-phase lower SMs**	**A-phase upper SMs**	**B-phase lower SMs**	**B-phase upper SMs**	**C-phase lower SMs**	**C-phase upper SMs**
Normal	100	0	0	0	0	0	0
A-phase lower SMs	0	96.3	0	2	0.3	0.8	1.3
A-phase upper SMs	0	0	98	0	0.4	0	0.5
B-phase lower SMs	0	1.5	0	97	0	1.8	0.1
B-phase upper SMs	0	0	1.8	0	97.2	0	3.8
C-phase lower SMs	0	1.5	0	1	1.2	96	0
C-phase upper SMs	0	0.7	0.2	0	0.9	1.4	94.3
**(c) Testing data proportion = 0.8**							
	**Normal**	**A-phase lower SMs**	**A-phase upper SMs**	**B-phase lower SMs**	**B-phase upper SMs**	**C-phase lower SMs**	**C-phase upper SMs**
Normal	100	0.1	0	0	0	0.2	0.4
A-phase lower SMs	0	96.1	0	1.4	0.7	0.8	2.4
A-phase upper SMs	0	0	94.8	0	2.1	0.1	1.8
B-phase lower SMs	0	2.5	0.7	96.1	0	2	1.4
B-phase upper SMs	0	0.1	1.6	0	92.6	0	2.7
C-phase lower SMs	0	0.6	1	2.5	1.2	96.4	1
C-phase upper SMs	0	0.6	1.9	0	3.4	0.5	90.3

**Table 7:** Detection accuracy of SoftMax.

Testing Data Proportion	0.1	0.2	0.3	0.4	0.5	0.6	0.7	0.8	0.9
Detection accuracy	100	100	100	100	100	100	100	100	100

**Table 8:** Sample confusion matrix of the classification results of the stand-alone SoftMax classifier.

**(a) Testing data proportion = 0.2**							
	**Normal**	**A-phase lower SMs**	**A-phase upper SMs**	**B-phase lower SMs**	**B-phase upper SMs**	**C-phase lower SMs**	**C-phase upper SMs**
Normal	100	0	0	0	0	0	0
A-phase lower SMs	0	98.5	0	0	0	0.5	0.5
A-phase upper SMs	0	0	100	0	0.2	0	0
B-phase lower SMs	0	0	0	100	0	0.8	0
B-phase upper SMs	0	0	0	0	99.8	0	0
C-phase lower SMs	0	1.5	0	0	0	98.5	0
C-phase upper SMs	0	0	0	0	0	0.2	99.5
**(b) Testing data proportion = 0.5**							
	**Normal**	**A-phase lower SMs**	**A-phase upper SMs**	**B-phase lower SMs**	**B-phase upper SMs**	**C-phase lower SMs**	**C-phase upper SMs**
Normal	100	0	0	0	0	0	0
A-phase lower SMs	0	98.1	0	0.4	0.2	0.8	0.4
A-phase upper SMs	0	0	99.4	0	0.7	0	0.2
B-phase lower SMs	0	0.7	0	99.6	0	0.6	0
B-phase upper SMs	0	0	0.6	0	99.1	0	0.6
C-phase lower SMs	0	0.6	0	0	0	97.4	0
C-phase upper SMs	0	0.6	0	0	0	1.2	98.8
**(c) Testing data proportion = 0.8**							
	**Normal**	**A-phase lower SMs**	**A-phase upper SMs**	**B-phase lower SMs**	**B-phase upper SMs**	**C-phase lower SMs**	**C-phase upper SMs**
Normal	100	0	0	0	0	0	0
A-phase lower SMs	0	97.1	0	1.5	0.4	1.4	3.7
A-phase upper SMs	0	0	95.6	0	1.3	0	0.5
B-phase lower SMs	0	2.2	0.3	96.6	0	2.3	0
B-phase upper SMs	0	0	1.5	0	94.3	0	2
C-phase lower SMs	0	0.5	0.3	1.9	0.8	95.9	0.7
C-phase upper SMs	0	0.2	2.3	0	3.2	0.4	93.1
